# Left Ventricular Trabeculations in Athletes: Epiphenomenon or Phenotype of Disease?

**DOI:** 10.1007/s11936-018-0698-8

**Published:** 2018-10-26

**Authors:** Mark Abela, Andrew D’Silva

**Affiliations:** 1 Cardiology Clinical Academic Group, St George’s, University of London, Cranmer Terrace, London, SW17 0RE UK; 2 MSc Sports Cardiology, Cardiology Clinical Academic Group, St George’s, University of London, London, UK

**Keywords:** Trabeculation, Noncompaction, Athlete’s heart, Exercise, Remodelling

## Abstract

**Purpose of review:**

Excessive trabeculation attracting a diagnosis of left ventricular noncompaction cardiomyopathy (LVNC) has been reported in ostensibly healthy athletes. This review aims to explain why this occurs and whether this represents a spectrum of athletic physiological remodelling or unmasking of occult cardiomyopathy.

**Recent findings:**

Genetic studies have yet to identify a dominant mutation associated with the LVNC phenotype and reported gene mutations overlap with many distinct cardiomyopathies and ion channel disorders, implying that the phenotype is shared across different genetic conditions. Large contemporary cohort studies indicate that current LVNC imaging criteria are oversensitive and not predictive of adverse clinical outcomes.

**Summary:**

The majority of excessive LV trabeculation, as assessed by current quantification methods, is not due to cardiomyopathy but forms part of the normal continuum in health with potential contributions from cardiac remodelling processes. The study of rare, severe LVNC phenotypes may yield insights into an underlying molecular pathogenesis but in the absence of a universally accepted definition, contamination with aetiologically distinct conditions expressing a similar phenotype will remain an issue. Automated, objective quantification of trabeculation will help to define the normal distribution using big data without the constraint of wide interobserver variation.

## Introduction

Left ventricular noncompaction cardiomyopathy (LVNC) is a condition characterised by a double-layered myocardial wall, possessing a thicker trabeculated layer with respect to compacted wall and deep intertrabecular recesses [1]. The archetypal clinical presentation consists of a triad of heart failure, ventricular arrhythmia, and systemic thromboembolism. Although presentations may be highly variable and individuals can be asymptomatic. Unfortunately, robust diagnostic criteria remain elusive. A reason for this is that the existence of LVNC as a distinct cardiomyopathy remains controversial with some favouring the hypothesis that excessive trabeculation is a phenotypic manifestation common to a variety of distinct myocardial processes and pathologies [2]. Some studies in populations of athletes have reported prominent trabeculation and this has raised concern over a potential diagnostic grey zone existing between LVNC and possible exercise-induced remodelling. In this review, we highlight the issues faced by the clinician when interpreting excessive left ventricular (LV) trabeculation in an athlete, more specifically, competing theories regarding the aetiopathogenesis of excessive LV trabeculation, issues with imaging over-diagnosis and a practical approach to investigating athletes.

When phenotypically severe cases are discovered in clinical practice, they add weight to the premise that LVNC could be a distinct pathological condition, particularly when the extent of trabeculae could not reasonably be achieved by adaptation alone. Human hearts are capable of remodelling driven by myocyte hypertrophy but the proliferative capacity of adult myocardial tissue is limited and therefore it is implausible that extensive trabeculation could be grown de novo. The vast majority of excessive LV trabeculation, however, is more likely to represent the wide spectrum of normal trabeculation and changes in appearance influenced by ventricular geometry and haemodynamic loading conditions.

## Origin of myocardial trabeculae and excessive LV trabeculation

Most publications have recycled a theory that LVNC results from an arrest of the normal foetal myocardial maturation where primitive, heavily trabeculated myocardial tissue fails to undertake the normal process of “compaction”, condensing to form the mature compact myocardial wall. This theory was based on intuitive observations as the disappearance of embryological trabeculae coincides with the development of a coronary circulation, and it was therefore presumed that the hypertrabeculation phase facilitated gas and nutrient exchange across a large surface area, permitting an increase in myocardial mass without constraint of diffusion from luminal blood. More recently, however, investigators have shown that this theory is incorrect and only the papillary muscles appearing to develop from previously trabeculated tissue. The compacted layer of the heart intrinsically proliferates and simply outgrows the extent of the trabeculae many fold [3, 4•].

## Genetic studies in LVNC

Our understanding of cardiomyopathies has been significantly advanced through the identification of gene mutations associated with cardiomyopathy phenotypes, providing insights into the underlying molecular morphogenesis. With respect to LVNC, no dominant genotype has emerged associated with excessive trabeculation; therefore, theories of a discrete molecular origin remain unsupported. Numerous gene mutations have been reported in LVNC phenotypes, which overlap with other cardiomyopathies and ion channelopathies. It is therefore argued that the LVNC phenotype may be a downstream consequence of disturbed molecular signalling and hence may result from a plurality of primary genetic disturbances in sarcomeric, cytoskeletal, mitochondrial, desmosomal, storage and ion channel proteins [5•, 6••, 7, 8]. A non-exclusive list of genes associated with LVNC is included in Table 1.

**Table 1:** Genes associated with left ventricular noncompaction cardiomyopathy

Gene	Protein	Inheritance	Associated conditions	Ref
ACTC1	Alpha cardiac actin	AD	HCM, DCM, ASD	5, 6, 8
DTNA	Alpha dystrobrevin	AD	DCM	5
LDB3	LIM domain binding 3/Cypher/ZASP	AD	HCM, DCM	5, 8
LMNA	Lamin A/C	AD	DCM	5, 8
MIB1	Mindbomb E3 ubiquitin protein ligase 1	AD	DCM	5
MYBPC3	Myosin binding protein C	AD	HCM, DCM	5, 8
MYH7	Beta-myosin heavy chain 7	AD	HCM, DCM	5
PRDM16	PR domain-containing protein 16	AD	DCM	5, 6, 8
TAZ G4.5	Taffazin	XL	Barth syndrome	5, 6, 8
TNNI3	Cardiac troponin I type 3	AD	HCM, RCM	8
TNNT2	Cardiac troponin T type 2	AD	DCM, HCM, RCM	5, 6, 8
TPM1	Alpha tropomyosin 1	AD	DCM, HCM	5, 8
mtDNA	Mitochondrial DNA dehydrogenase subunit 1; ATPase subunit 6 and 8	Matrilineal	Mitochondrial cytopathies	5
TTN	Titin	AD	DCM	6
DES	Desmin	AD	DCM, RCM	6
DSP	Desmoplakin	AD	DCM	6
FKTN	Fukutin	AD	DCM, skeletal myopathy	6
HCN4	Potassium/sodium hyper polarising-activated cyclic nucleotide-gated channel 4	AD	MVP, bradycardia, Brugada syndrome	6, 8
KCNQ1	Potassium voltage-gated channel subfamily Q1	AD	LQTS	6
LAMP2	Lysosomal associated membrane protein 2	XL	Danon disease	6
NOTCH1	Notch 1	AD	DCM	6
PLN	Phospholamban	AD	HCM, DCM	6, 8
RYR2	Ryanodine receptor 2	AD	CPVT	6
SCN5A	Sodium voltage-gated channel alpha subunit 5	AD	Brugada syndrome, LQTS, DCM, AF, SSS	6
CASQ2	Calsequestrin 2	AR	CPVT	8
OBSCN	Obscurin	AD	HCM, DCM	7

*AD*, autosomal dominant; *AF*, atrial fibrillation; *AR*, autosomal recessive; *ASD*, atrial septal defect; *CPVT*, catecholaminergic polymorphic ventricular tachycardia; *DCM*, dilated cardiomyopathy; *HCM*, hypertrophic cardiomyopathy; *LQTS*, long QT syndrome; *MVP*, mitral valve prolapse; *RCM*, restrictive cardiomyopathy; *SSS*, sick sinus syndrome; *XL*, X-linked

A large multi-centre study of 327 predominantly adult patients with an LVNC phenotype reported on comprehensive genetic analysis. Firstly, they found that the group could be divided into those with causative genetic mutations comprising 32%, those with a familial cardiomyopathy but no causative mutation comprising 16% and sporadic cases, with neither a family history nor gene mutation, comprising 52%. Secondly, of the genetic causes identified, 71% were accounted for by mutations in genes MYH7, MYPC3 and TTN [6••]. Studies including paediatric populations report a higher genetic yield compared to adults but there is considerable heterogeneity [6••, 9, 10]. A contemporary study of 128 paediatric “LVNC cases” found that 59% were sporadic cases and the yield of genetic testing was only 9%, lower than other genetic studies. The study highlights important differences between paediatric and adult populations, where 9% had an underlying metabolic or syndromic genetic condition and the proportion of cases with overlapping cardiomyopathies or cardiovascular malformations was considerable (52%) [11•]. It is important to consider that rare variants of unknown significance might be reclassified in the future as likely pathogenic, and similarly, others thought likely pathogenic currently may be found to be benign. These genetic studies potentially support a greater role of environmental remodelling or epigenetic contributions to LVNC, owing to the majority appearing sporadic, and the association with genetic syndromic conditions or other cardiomyopathy gene mutations suggests that multiple diverse pathologies can produce a similar phenotype.

## Potential role of increased trabeculae in cardiac adaptation

Why should increased trabeculation offer an adaptive advantage? Halaney et al. studied six explanted hearts, and through the use of finite-element model simulations analysed the effects of cutting away the trabeculae on LV compliance. They found that whilst an absence of trabeculae offers the greatest LV compliance, the apex is subjected to high wall stress and strain. The presence of apical trabeculae redistributes apical wall stress, potentially protecting the thin-walled apex from adverse aneurysmal remodelling [12]. Paun et al. studied the effect of LV trabeculations on ventricular performance and size using computational modelling. They demonstrated that in a smooth, non-trabeculated LV, increases in stroke volume came at considerable energetic cost, requiring increasingly high longitudinal strain, whereas a trabeculated LV was capable of achieving high stroke volumes without incurring exponential elevations in strain [13]. Both of these studies, however, have important limitations in mathematical oversimplification of left ventricular geometry, viscoelastic properties of trabeculae and myocardial deformation.

The contribution of LV rotation and twist remains a relatively understudied aspect of myocardial function considering its pivotal role in the mechanical efficiency of the heart, both in ejection and diastolic suction. van Dalen et al. have demonstrated using echocardiography that patients with excessive trabeculation, suspected as having LVNC, demonstrate near-absent apical twist and LV solid body rotation, with basal and apical rotation in the same direction [14, 15]. The investigators came to the logical conclusion that if LVNC is the persistence of embryological trabeculated myocardium, normal three-layered fibre orientation may be disturbed in the compacted myocardium. Alternatively, primary dysfunction in the ability of the LV to twist, during a critical period in development, could potentially trigger compensatory hypertrabeculation in an effort to facilitate ventricular emptying whilst conserving myocardial energy. Theoretically, this would allow multiple diverse myocardial pathologies to produce LV hypertrabeculation as a compensatory mechanism for dysfunctional LV twist. Future studies utilising diffusion tensor cardiac magnetic resonance (CMR) in subjects with excessive trabeculation could yield valuable insights if myocardial sheetlet orientations are disturbed [16].

## LVNC imaging criteria and their limitations

It remains a clinical challenge to define LVNC in the absence of accepted diagnostic criteria, or in fact appreciate how much LV trabeculation is considered excessive.

In an effort to address these issues and identify cases, several investigators have proposed imaging criteria for the diagnosis of LVNC. To date, numerous criteria have been published across various imaging modalities [14, 17–33] shown in Table 2; however, none have demonstrated adequate sensitivity and specificity when externally validated in large cohorts, generating a huge potential for false-positive diagnoses if applied indiscriminately.

The first echocardiographic criteria were proposed by Chin et al. [17] are also known as the California criteria [34]. These were based on a case series of eight, predominantly paediatric patients and generated a Chin X/Y ratio, measuring the epicardial distance to the trabeculation trough against the epicardial distance to the trabeculation peak in a long-axis, diastolic view, though many have subsequently applied this method to short-axis images.

Jenni et al. proposed the second set of criteria [18], also known as the Zurich [34] or Swiss criteria [35]. The investigators highlighted that measurement was often difficult in diastole and the boundary between myocardial layers more easily distinguished in systole. These were derived from 34 adult patients, collected over a 13-year period with 7 having histopathological examination providing anatomical correlation. Moreover, rather than comparing “LVNC cases” with healthy controls, patients harbouring other disease pathologies associated with prominent trabeculations (dilated cardiomyopathy and hypertensive LV hypertrophy) were used for comparison. A maximal, end-systolic, short-axis trabeculated layer thickness at least twice that of the adjacent compacted layer was felt to discriminate LVNC from other pathologies. In addition to this 2:1 ratio, it was emphasised that additional conditions must be met for the diagnosis of “isolated LVNC”, including the absence of any coexisting cardiac anomaly, and that colour Doppler imaging must demonstrate that blood from the ventricular cavity perfuses the intertrabecular recesses directly.

Stollberger et al. [19] proposed a third set of criteria, also known as the Vienna criteria [34]. Acknowledging that end-systolic, short-axis images employed in the Jenni criteria are vulnerable to erroneous inclusion of papillary muscles, aberrant bands and false tendons in the trabecular layer, the investigators favoured long-axis, end-diastolic images for measurement. Stollberger et al. also undertook a different approach, instead of drawing upon collected “LVNC cases” to define what is “abnormal”, they began by utilising autopsy data to define the extent of normal trabeculation in human hearts. Of 474 hearts studied previously, 68% showed prominent trabeculation but only in 4% could more than three trabeculae be counted apically to the papillary muscles, with no heart possessing more than five trabeculae [36]. The Stollberger criteria moved away from “isolated LVNC” to favour “LV hypertrabeculation (LVHT)”, aiming to include cases with additional cardiac anomalies and stipulated that (1) > 3 trabeculations protruding from the LV wall, apically to the papillary muscles, visible in one imaging plane and (2) perfusion of intertrabecular spaces from the ventricular cavity visualised on colour Doppler were required for the diagnosis. The investigators originally resisted the inclusion of a trabeculated to compacted layer ratio, taking the view that any ratio could be compatible with the diagnosis of LVNC [35, 37, 38]; however, they later incorporated the end-systolic 2:1 ratio from the Jenni criteria [39••]. Despite amalgamation of echocardiographic criteria, expert observers, with 20–29 years’ experience in assessing LVHT, found low interobserver agreement with disagreement in 35% of cases, which even after mutual review remained unresolved in 11% [39••].

Even when assessed separately, echocardiographic criteria measuring trabeculation do not identify the same cases and show poor correlation [40, 41], which is unsurprising as measurements are made in different imaging planes, at different anatomical locations and in different phases of the cardiac cycle.

Considering that when the commonly used echocardiographic LVNC criteria were originally created, the images used were acquired on machines only capable of fundamental imaging with lower resolution and therefore less able to visualise trabeculae and the border between myocardial layers. Current routine use of harmonic imaging may contribute to the epidemic of positive LVNC imaging criteria, which would have previously eluded detection.

Echocardiography occasionally has difficulty visualising trabeculation in some patients with challenging body habitus or anatomy, and more accurate assessment of the cardiac apex can be provided with cross-sectional imaging techniques, unrestricted by acoustic windows or near-field clutter artefact. CMR is a valuable complimentary imaging technique, providing additional tissue characterisation through scar imaging in suspected cardiomyopathy cases.

CMR has seen a similar proliferation in LVNC criteria. Petersen et al. proposed the first, which remains one of the most frequently used in clinical practice due to the ease of measurement without the need for additional analysing software. Similar to the Jenni criteria, the investigators compared 7 “LVNC cases” with a variety of conditions associated with prominent trabeculations, including athletes, hypertrophic cardiomyopathy, dilated cardiomyopathy, hypertensive heart disease and aortic stenosis patients in addition to healthy controls. They concluded that a trabeculated to compacted layer ratio of > 2.3, measured in an end-diastolic long axis view but excluding the apex (segment 17), provided the best sensitivity and specificity for identifying the “LVNC cases” [26]. They were also aware of the potential for over-diagnosis if applying this threshold to a population with a low pre-test probability of cardiomyopathy, suggesting that the findings were only useful in the context of clinical suspicion such as heart failure, arrhythmia, systemic thromboembolism, family history of LVNC, associated muscular disorders or regional wall motion abnormalities [42].

Jacquier et al. proposed an alternative, more comprehensive way of measuring trabeculation through drawing contours in the entire short-axis stack both including and excluding trabeculated myocardium and calculating the difference. This method generates a trabecular mass percentage as a proportion of the total LV mass. A value of over 20% was shown to discriminate well between “LVNC cases”, dilated cardiomyopathy, hypertrophic cardiomyopathy cases and healthy controls [27]. Though this method best represents what is fundamentally a continuous variable and includes measurements across the entire LV, a number of criticisms of this technique have been highlighted. Principally, the low interobserver reproducibility reported with this method [43], perhaps emanating from differences in analytical software and precision of contouring between observers, who may adjudicate differently as to what constitutes the papillary muscle mass. Many examples of this are evident when other groups testing new LVNC criteria raise the percentage trabeculated LV mass threshold to 25% [29], 35% [32] and even as high as 40% [31]. Various reasons are given for these adjustments including better receiver-operator curve characteristics and inclusion of papillary muscle mass.

A novel method of quantifying trabeculation proposed by Captur et al. employs automated calculation of fractal dimensions of the endocardial boarder as a measure of endocardial complexity [30]. Though the method requires analysis of each endocardial contour in the short axis stack, the investigators proposed that a maximal apical fractal dimension of > 1.3 had the best receiver-operator curve characteristics in the derivation cohort, and a mean global fractal dimension of > 1.26 was reasonable at differentiating “LVNC cases” from control subjects of black and white ethnicities. However, when this method was applied to a large, healthy, multi-ethnic cohort, 21% possessed an apical fractal dimension > 1.3 [44]. Unlike other methods of trabecular quantification, fractal dimension ignores the thicknesses of the trabeculated and compacted myocardial layers, which may conceivably lower its concordance with other methods of trabecular quantification.

The criteria listed in Table 2 represent only some of the proposed methods of quantifying abnormal trabeculation and attempting to diagnose LVNC. Applying all these criteria to clinical cases is not only impractical but also increases the likelihood that at least one method will be positive when others are not, generating uncertainty in the interpretation.

**Table 2:** Summary of diagnostic imaging criteria for left ventricular noncompaction cardiomyopathy

Modality	First author	Year and journal	Patients (No.)	Measurement	Imaging plane	Cardiac phase
Echo	Chin et al.(California criteria) [17]	1990 Circulation	8 (7 children)	X:Y ≤ 0.5	LAx	ED
	Jenni et al. (Zurich or Swiss criteria) [18]	2001 Heart	34	N:C ≥ 2	SAx	ES
	Stollberger et al. (Vienna criteria) [19]	2002 Am J Cardiol	62	> 3 trabeculae (± N:C ≥ 2)	LAx (± SAx)	ED (±ES)
	Pignatelli et al. [20]	2003 Circulation	36 children	N:C > 1.4	LAx	ED
	Belanger et al. [21]	2008 Am J Cardiol	60	N:C > 2 + area > 5 cm^2^	LAx + SAx	ES
	van Dalen et al. [14]	2008 Eur J Heart Fail	10	LV twist-solid body rotation	SAx	Systole and diastole
	Paterick et al. [22] (Milwalkee criteria)	2012 Circ J	0 (no validation)	N:C ≥ 2	SAx	ED
	Ghebhard et al. [23]	2012 J Am Soc Echocardiogr	41	Max systolic C layer < 8.1 mm	SAx	ES
	Yubbu et al. [24]	2018 Int J Cardiol	30 children	Circumferencial strain <− 24.5%	SAx apical	Systole and diastole
3D-Echo	Caselli et al. [25]	2012 J Am Soc Echocardiogr	17	TV > 15.8 ml or TV% > 12.8%	LAx	ED
CMR	Petersen et al. [26]	2005 J Am Coll Cardiol	7	N:C > 2.3	LAx	ED
	Jacquier et al. [27]	2010 Eur Heart J	16	TM > 20%total	SAx	ED
	Fazio et al. [28]	2010 Int J Cardiol	1	N:C > 2.5	SAx	ED
	Grothoff et al. [29]	2012 Eur Radiol	12	TM >25% and N:C ≥ 3	SAx + LAx	ED
	Captur et al. [30]	2013 J Cardiovasc Magn Reson	30	Global FD > 1.26	SAx	ED
				Apical FD > 1.3		
	Stacey et al. [31]	2013 J Am Coll Cardiol Img	122	N:C > 2	SAx	ES
	Choi et al. [32]	2016 J Cardiovasc Magn Reson	54	TV > 35% total	SAx	ED
CT	Melendez-Ramirez et al. [33]	2012 J Cardiovasc comput Tomogr	10	N:C > 2.2	SAx	ED

*C*, compacted layer thickness; *CMR*, cardiac magnetic resonance; *CT*, computed tomography; *Echo*, echocardiography; *ED*, end-diastole; *ES*, end-systole; *LAx*, long-axis view; *N*, non-compacted layer thickness; *SAx*, short-axis view or stack; *TV*, trabeculated left ventricular volume; *TV%*, percentage trabeculated left ventricular volume as a proportion of the left ventricular end-diastolic cavity; *X*, distance of epicardial layer to trabecular trough; *Y*, distance of epicardial layer to the trabecular peak

## Performance of imaging criteria in large cohorts

Any of the LVNC criteria when applied to large healthy or heart failure populations generate sizeable false positive results. Kohli et al. examined echocardiograms of patients referred for heart failure, finding that 24% fulfilled one or more criteria for LVNC [40]. Zemrak et al. examined 2742 CMR studies from the American Multi-Ethnic Study of Atherosclerosis (MESA) cohort, demonstrating that 26% satisfied Petersen’s LVNC criteria. Intriguingly, this population were re-examined over a 9.5-year follow-up period which reassuringly showed that increased trabeculation in this healthy population was not associated with adverse outcomes, such as heart failure, arrhythmia or systemic embolism [45]. Over the last 4 years, there has been a proliferation of similar, large, cohort studies reaching similar conclusions relating to potential for imaging over-diagnosis, poor concordance between techniques and a lack of association between trabeculation severity and adverse clinical outcomes [46••, 47, 48•, 49•], summarised in Table 3.

**Table 3:** Summary of large cohort studies investigating the prevalence and clinical significance of excessive left ventricular trabeculation using cardiac magnetic resonance imaging

First author and journal	Year	Patients (No.)	Population	Criteria used	Average follow-up	Summary of findings
Zemrak et al. [45] J Am Coll Cardiol	2014	2742	MESA study, multisite USA (healthy)	Petersen	9.5 years	25.7% fulfilled Petersen’s criteria in at least 1 segment.
						No association with adverse cardiovascular outcomes.
Amzulescu et al. [47] J Am Coll Cardiol Img	2015	162 + 48	NI-DCM patients for CMR and healthy controls, single centre, Belgium	Petersen Jacquier (modified)	3.4 years	Petersen’s criteria met in 36% and modified Jacquier’s criteria in 44%.
						No association with major adverse cardiovascular events.
Weir-McCall et al. [46••] J Am Coll Cardiol Img	2016	1480	TASKFORCE study, multi-site UK (healthy)	Petersen	Snapshot, no follow-up	14.8% fulfilled one criteria, 7.9% two criteria, 4.3% three criteria, 1.3% all four criteria have poor specificity for LVNC.
				Stacey		
				Grothoff		
				Jacquier		
Andreini et al. [48•] J Am Coll Cardiol	2016	113	Echo diagnosis of LVNC, multi-national	Petersen (modified)	2 years	Extent of trabeculation had no prognostic influence over LV dilatation, systolic dysfucntion and LGE presence.
				Jacquier (modified)		
Ivanov et al. [49•] Circ Cardiovasc Imaging	2017	700	Consecutive patients refered for CMR, single centre, USA	Petersen	7 years	Petersen’s criteria fulfilled in 39% Stacey’s in 23%, Jacquier’s 25%, Captur’s in 3%.
				Stacey		
				Jacquier		
				Captur		
						No association with cardiac death, stroke, ventricular arrhythmia or heart failure hospitalisation.

*LGE*, late gadolinium enhancement; *LV*, left ventricular; *LVNC*, left ventricular noncompaction cardiomyopathy, *NI-DCM*, non-ischaemic dilated cardiomyopathy; *UK*, United Kingdom; *USA*, United States of America

In an effort to distinguish LVNC from normality, the dichotomous diagnostic threshold grossly oversimplifies the natural complexity of trabeculation, including its variability. Attempts to generate such thresholds are hampered by multiple sources of bias. Spectrum bias describes how diagnostic thresholds have been derived from very small representations of normal or “diseased” states, when the phenotypes of both groups are in reality quite varied. The small numbers of cases included in derivation cohorts are unable to provide an accurate representation of all of the phenotypic manifestations in the respective groups. When diagnostic thresholds are applied to a real-world setting, they perform poorly because disease and non-diseased spectrums are inadequately represented, resulting in inability to distinguish between mild disease and the extremes of normality [50•]. Due to the absence of a gold standard, the test being studied becomes incorporated into the gold standard, referred to as incorporation bias [50•]. When “LVNC cases” are determined by imaging characteristics and subsequently imaging tools are employed to establish accuracy in “diagnosis”, incorporation bias exaggerates the sensitivity and specificity. Ascertainment and publication biases heavily influence the current impressions of the natural history, clinical presentation and prognosis of “LVNC”.

## Excessive trabeculation in athletes

Athletes engage in intensive physical activity on a regular basis resulting in a constellation of physiological, structural and electrical adaptations on the cardiovascular system, collectively referred to as the “athlete’s heart”. Typical remodelling changes visualised by echocardiography include 15–20% greater LV wall thickness and 10% larger biventricular cavity sizes with respect to sedentary controls [51–54]. These adaptations permit rapid filling and augmentation of stroke volume to deliver a large and sustained increase in cardiac output to active muscles for prolonged periods. On occasion, these physiological adaptations overlap with morphological mild cardiomyopathies, though the distinction is an important one as sudden cardiac death is the most common cause of death in athletes and in those under 35 years of age, rare inherited or congenital conditions account for the majority of deaths [55, 56]. With this in mind, many asymptomatic athletes are screened for underlying cardiovascular conditions, and their investigation results are studied to provide a more detailed understanding of the athlete’s heart across different sporting disciplines, ethnicities, ages and genders. One such study utilising observational data from screening investigated the prevalence of excessive trabeculation in over a thousand athletes and found that 8.1% tested positive for LVNC by both Chin and Jenni criteria, compared with 7.0% in 415 sedentary controls [57]. Another similar study was performed including over 2500 Olympic athletes and found that only 1.4% demonstrated excessive trabeculation potentially suggestive of LVNC. Furthermore, based on clinical evaluation including symptoms, family history, LV systolic and diastolic function, arrhythmias and CMR findings, the investigators suggested that only 0.1% were considered to have LVNC with the remainder being either normal or unlikely to harbour cardiomyopathy [1].

As the majority of athletes with prominent trabeculation demonstrate no other cardiomyopathic features, it has been proposed that excessive trabeculation may be an exercise-induced remodelling phenomenon. A longitudinal study of 102 primagravida pregnant women tested a similar remodelling hypothesis [41]. Pregnancy is associated with a 40% increase in blood volume and cardiac output, resulting in a 10–20% increase in LV mass [58]. Based on these naturally occurring cardiovascular remodelling changes, Gati et al. investigated whether dynamic changes in LV trabeculation also occur in pregnancy and found that in the third trimester of pregnancy, 19% satisfied the Chin criteria, 10% satisfied the Jenni criteria and 8% satisfied both, where none had demonstrated excessive trabeculation in the first trimester. Moreover, this increased LV trabeculation resolved within 24 months of follow-up in 73%, with marked regression in the remainder [41]. To date, there have been no prospective longitudinal studies investigating the development of increased LV trabeculation in exercising individuals.

A case series of four professional basketball players with excessive LV trabeculation were evaluated with echocardiography at the beginning of the training season, during training and after a period of detraining. These athletes developed increased trabeculation, which was reportedly most pronounced in the peak-training period and associated with athletic ECG repolarization changes [59]. The series was limited by the small size and lack of trabeculation quantification, though highlights the risk of LVNC overdiagnosis in a low risk population with a likely alternative mechanism for excessive trabeculation than cardiomyopathy. A larger case series comprehensively investigated 22 asymptomatic athletes with excessive trabeculation and sub-divided them into two groups. Group A consisted of 11 subjects with LV ejection fraction (EF) < 55% and/or LV end-diastolic dimension (LVEDD) > 60 mm and the remainder with LVEF ≥ 55% and LVEDD ≤ 60 mm were included in group B. Though group A contained single cases of left-bundle branch block and non-sustained ventricular tachycardia, other parameters suggested an athletic eccentric remodelling phenotype, without convincing evidence that this multi-parametric strategy could provide a clear distinction between physiological remodelling and pathological LVNC [60].

## Management of athletes with excessive trabeculation

When excessive trabeculation is incidentally found on imaging, a pragmatic, multi-modality approach is prudent in assessing for possible indicators of cardiomyopathy. Features in the history supporting cardiomyopathy include symptoms of heart failure, syncope, palpitation, neurological/neuromuscular symptoms, thromboembolic events or a family history of cardiomyopathy. Conduction disease such as left bundle branch block or repolarisation abnormalities such as pathological T wave inversions are similarly suggestive of cardiomyopathy. Ventricular arrhythmias, either on ambulatory monitoring or exercise testing as well as functional testing abnormalities including a low peak oxygen consumption (VO_2_) on cardiopulmonary exercise testing or a failure to increase LVEF by at least 11% on peak exercise imaging [61] potentially indicate disease. Specific additional imaging parameters including moderate or severe LV systolic impairment, diastolic dysfunction, regional wall motion abnormalities and late gadolinium enhancement on CMR have also been proposed as discriminatory features favouring pathology.

**Fig. 1:**
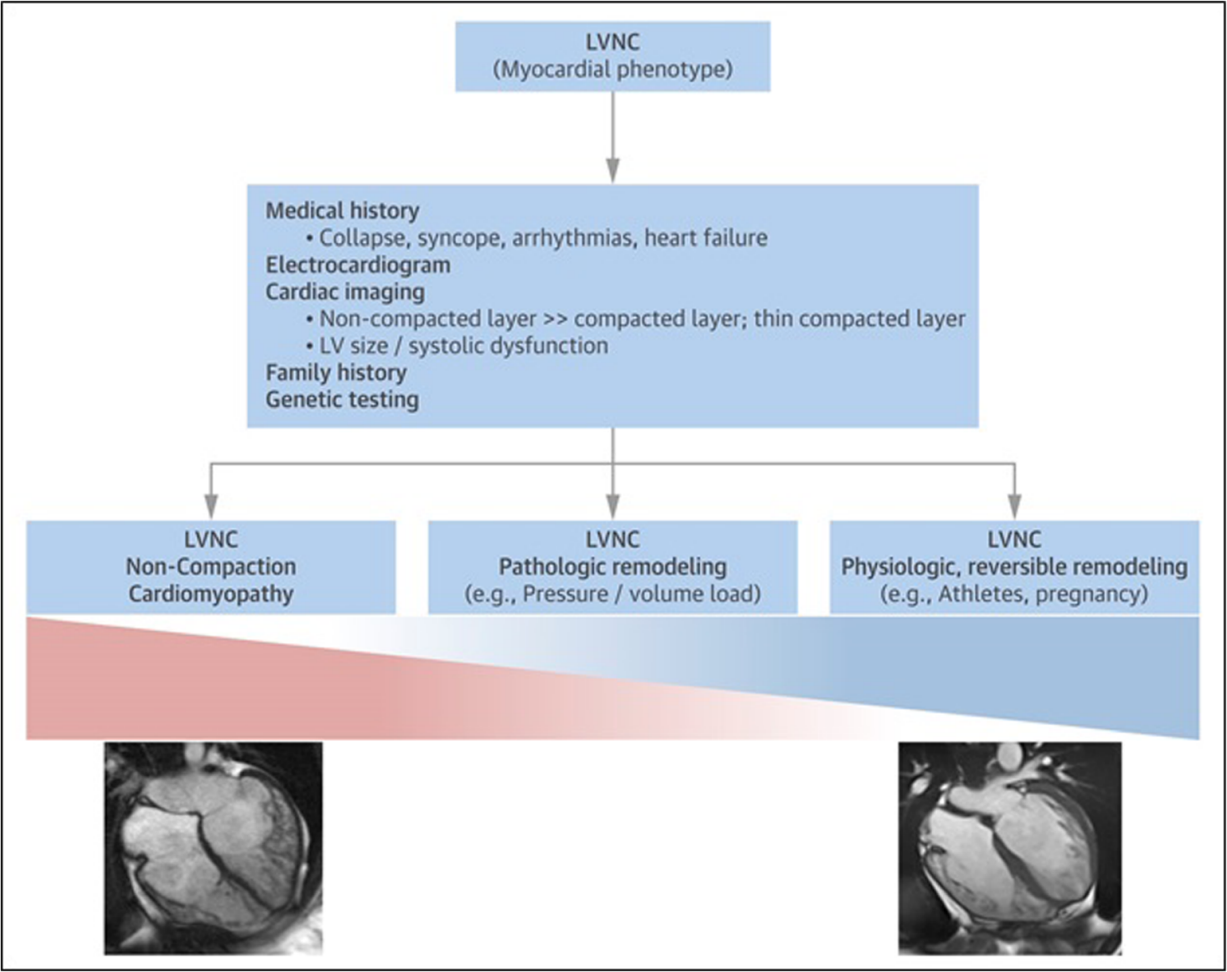
Myocardial phenotype of LVNC as the expression of a spectrum of different underlying pathophysiological mechanisms and proposed systematic clinical examination. Figure adapted with permission from Oechslin et al. [62]. LVNC, left ventricular noncompaction.

Such schema offer a practical starting point [62](see Fig. 1); however, ambiguous cases will arise where dual pathologies are possible. Pragmatically, the presence of trabeculation in combination with other cardiomyopathy phenotypes, such as dilated cardiomyopathy, does not add additional prognostic information nor influence management. The care of individuals with suspected LVNC follows the same principles as dilated cardiomyopathy, which may include treatment of heart failure symptoms and LV systolic dysfunction, arrhythmia management, anticoagulation for those suffering thromboembolic events or atrial fibrillation, cardiac resynchronisation and defibrillator devices if indicated, advanced cardiac support including transplantation where appropriate and family screening.

With respect to the care of athletes, contemporary recommendations from the American Heart Association advise that competitive sports may be considered for asymptomatic athletes with excessive LV trabeculation, provided that there is no prior history of syncope and important ventricular tachyarrhythmias on ambulatory monitoring or exercise testing are excluded. Athletes discovered to have cardiomyopathic features such as impaired LV systolic function, atrial or ventricular tachyarrhythmias or a history of syncope are advised not to participate in competitive sports with the possible exception of low-intensity class 1A sports [63••].

## Conclusion

At the present time, little progress has been made in our understanding of LVNC when compared to other cardiomyopathies. Fundamental questions remain unanswered, specifically, what is the upper limit of normal LV trabeculation? Is LVNC a distinct cardiomyopathy and if so, how should it be diagnosed and what is the natural history of the condition? LVNC studies are likely to contain heterogeneous populations with mixtures of distinct diseases and normal individuals with prominent trabeculation. Greater discipline in the diagnosis of LVNC must be exercised and this should not be purely based on imaging. Those who believe that the phenotypically severe cases represent “true LVNC” should seek better definition with consensus Task Force criteria potentially incorporating clinical, familial and additional morphological information. Given that such phenotypically severe cases will be rare and case definition more restrictive, international registries will be required and more elaborate classification systems such as the MOGE(S) nosology [64] may be useful in providing uniformly detailed phenotyping.

Technological advancements offer important improvements, as fully automated methods of trabeculation quantification are being developed and refined. These approaches can reduce interobserver variation, be scalable to interrogate large population datasets and biobanks to define normative values and may permit the investigation of complex relationships with LV geometry, strain, wall stress and loading conditions.

Diagnostic imaging criteria require rationalisation as collectively they result in unacceptable false-positive results, over-investigation, unnecessary treatment and potential loss of opportunity in the case of athletes. In the present era, the discovery of excessive trabeculation should be approached with curiosity as to its cause and caution in interpreting its clinical significance.
